# SNAI1 and SNAI2 Are Asymmetrically Expressed at the 2-Cell Stage and Become Segregated to the TE in the Mouse Blastocyst

**DOI:** 10.1371/journal.pone.0008530

**Published:** 2009-12-31

**Authors:** Christine E. Bell, Andrew J. Watson

**Affiliations:** 1 Department of Obstetrics and Gynaecology, The University of Western Ontario, London, Ontario, Canada; 2 Department of Physiology and Pharmacology, The University of Western Ontario, London, Ontario, Canada; 3 Department of Medicine, The University of Western Ontario, London, Ontario, Canada; 4 Children's Health Research Institute, The University of Western Ontario, London, Ontario, Canada; 5 Lawson Health Research Institute, London, Ontario, Canada; Ottawa Hospital Research Institute and University of Ottawa, Canada

## Abstract

SNAI1 and SNAI2 are transcription factors that initiate Epithelial-to-Mesenchymal cell transitions throughout development and in cancer metastasis. Here we show novel expression of SNAI1 and SNAI2 throughout mouse preimplantation development revealing asymmetrical localization of both SNAI1 and SNAI2 in individual blastomeres beginning at the 2-cell stage through to the 8-cell stage where SNAI1 and SNAI2 are then only detected in outer cells and not inner cells of the blastocyst. This study implicates SNAI1 and SNAI2 in the lineage segregation of the trophectoderm and inner cell mass, and provides new insight into these oncogenes.

## Introduction

Preimplantation development is characterized by the differentiation of two distinct cell types, inner cell mass (ICM) which will form the embryo proper and the trophectoderm (TE) which will contribute to the embryonic portion of the placenta [1]. The TE is a polarized, epithelial cell type and the development of the TE is the first differentiation event to occur throughout mammalian development. The cell polarity model proposes that TE differentiation is initiated at the 8-cell stage and that TE cell fate occurs through the establishment of cell polarity along the length of each blastomere forming an apical and basolateral membrane [1]. If a blastomere undergoes symmetric division, each daughter cell inherits equally the apical and basolateral membrane, will remain polarized, and contribute to the TE [1]. If a blastomere undergoes asymmetric division, each daughter cell inherits a distinct portion of the polarized membrane, either apical and basolateral or just basolateral, resulting in the differentiation of a TE cell and an ICM cell respectively [1]. Prior to the compacted 8-cell stage in the mouse however, blastomeres are totipotent and can contribute to all cell types of the blastocyst [2]. The outer embryonic blastomeres that do go on to form the TE acquire several critical gene products such as Na/K-ATPase [3]–[5], tight junctions [6] and adherens junction, aquaporins [7], and several transcription factors, such as caudal homeobox 2 (CDX2) [8] which define the TE cell lineage and orchestrate its function during blastocyst formation.

Recently, Na/K-ATPase β1 subunit expression has been shown to be regulated in cell lines by the two TFs, SNAI1 (previously known as SNAIL) and SNAI2 (previously known as SLUG) [9]. SNAI1 expression was increased in MDCK cells resulting in the down-regulation of the Na/K-ATPase β1. E-cadherin expression was also down-regulated resulting in the loss of cell-cell contacts [9]. Conversely, SNAI2 expression promoted Na/K-ATPase β1 subunit expression by inhibiting SNAI1 expression [9]. Aberrant expression of SNAI1 [10]–[13] and SNAI2 [13], [14] is also linked to cancer metastasis in epithelial cell lines through its role in directing epithelial-to-mesenchymal cell transition (EMT). SNAI1 and SNAI2 direct the loss of polarity in epithelial cells by down-regulating E-cadherin expression resulting in EMT. Once EMT has occurred, a cell is no longer adherent to its neighbors and re-engages cell proliferation and metastasis programs [13]. *Snai1* knock-out studies in mice have revealed a role for SNAI1 in neural crest differentiation [10]. *Snai2* knock-out mice do not display an embryonic lethality and survive to birth [15]. Since SNAI1 and SNAI2 are important mediators of E-cadherin and Na/K ATPase β1 subunit expression, (necessary components for TE differentiation and maintenance), we have characterized *Snai1* and *Snai2* expression throughout mouse preimplantation development and TE differentiation. Our results have revealed a novel and unexpected protein localization pattern for SNAI1 and SNAI2 in the 2-cell and 4-cell embryo and have demonstrated that SNAI1 and SNAI2 become confined solely to the outer cells, TE cell lineage of the early embryo and are not present in the inner cell, ICM lineage of the embryo.

## Results

### Quantitative RT-PCR of Snai1 and Snai2 throughout Preimplantation Development

**Figure 1:**
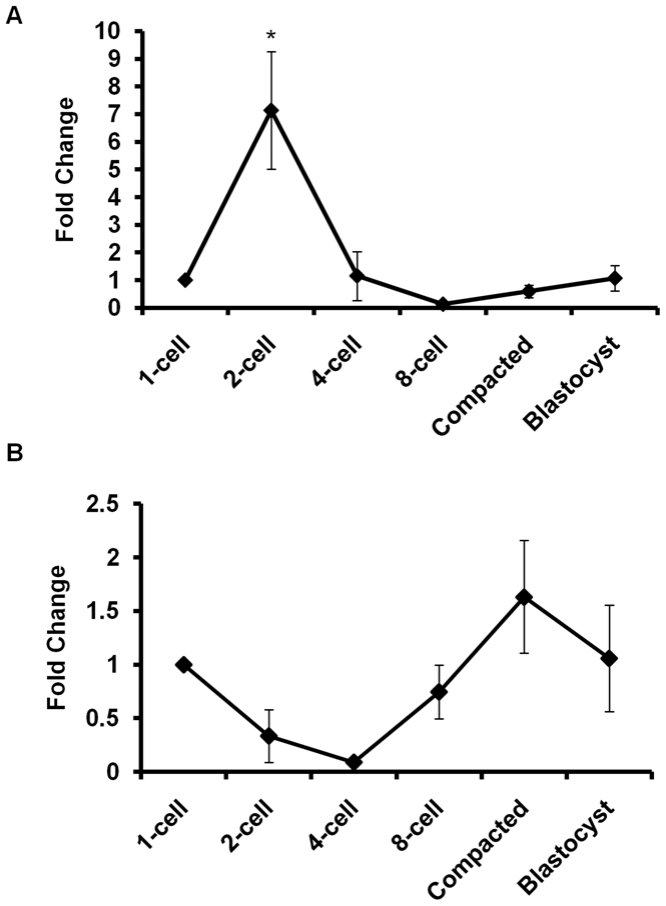
Characterization of *Snai1* and *Snai2* expression. (A) *Snai1* transcripts were detected using quantitative RT-PCR performed on pools of 20 fresh flushed embryos, at each stage of development (1-cell, 2-cell, 4-cell, 8-cell, compacted embryo and blastocyst); n = 3, p<0.05±S.E.M. (B) *Snai2* transcripts were detected using quantitative RT-PCR performed on pools of 20 fresh flushed embryos, at each stage of development (1-cell, 2-cell, 4-cell, 8-cell, compacted embryo and blastocyst); n = 3, p<0.05±S.E.M.

**Figure 2:**
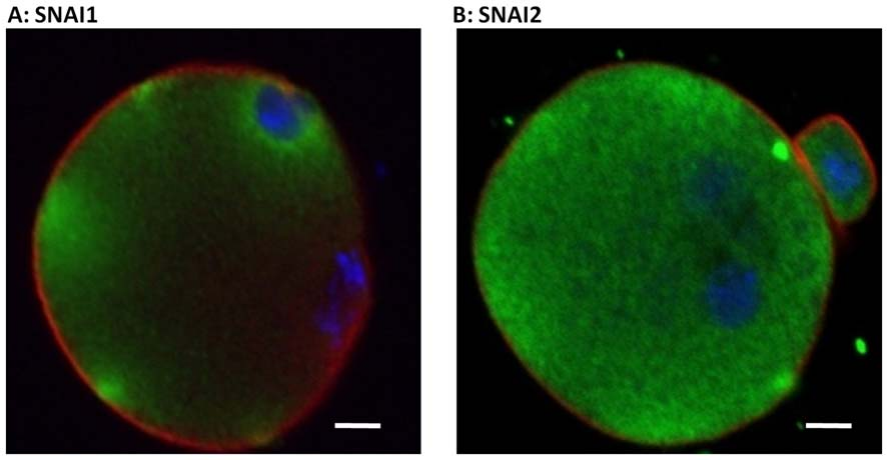
Localization of SNAI1 and SNAI2 in the 1-cell Zygote. (A) SNAI1 localization appears in discrete fluorescent foci in cortical regions throughout the zygote; n = 9. (B) SNAI2 localization is symmetrical however, there are areas with increased intensity throughout the zygote; n = 10. Red =  Filamentous Actin; Blue = Nuclei; Green  =  SNAI1 or SNAI2. Scale bars  = 10 µM.

When quantitative RT-PCR was performed on each developmental stage (1-cell, 2-cell, 4-cell, 8-cell, compacted embryo, blastocyst), it revealed that *Snai1* and *Snai2* transcripts were differentially regulated throughout preimplantation development (Figures 1A and 2A respectively). *Snai1* transcripts were detected as early as the 1-cell stage, and then *Snai1* was significantly up-regulated at the 2-cell stage followed by down regulation at the 8-cell stage and return to baseline levels at the blastocyst stage (Figure 1A). Detection of *Snai1* throughout preimplantation development confirms previously reported detection by Veltmaat et al., 2000, however, here we report relative levels of *Snai1.*

*Snai2* transcripts were also detected as early as the 1-cell stage*;* however *Snai2* transcripts were down regulated at the 2-cell stage and did not re-accumulate until the 8-cell stage, and increased up to the blastocyst stage (Figure 1B).

### Whole Mount Immunofluoresence of SNAI1 and SNAI2 throughout Preimplantation Development

**Figure 3:**
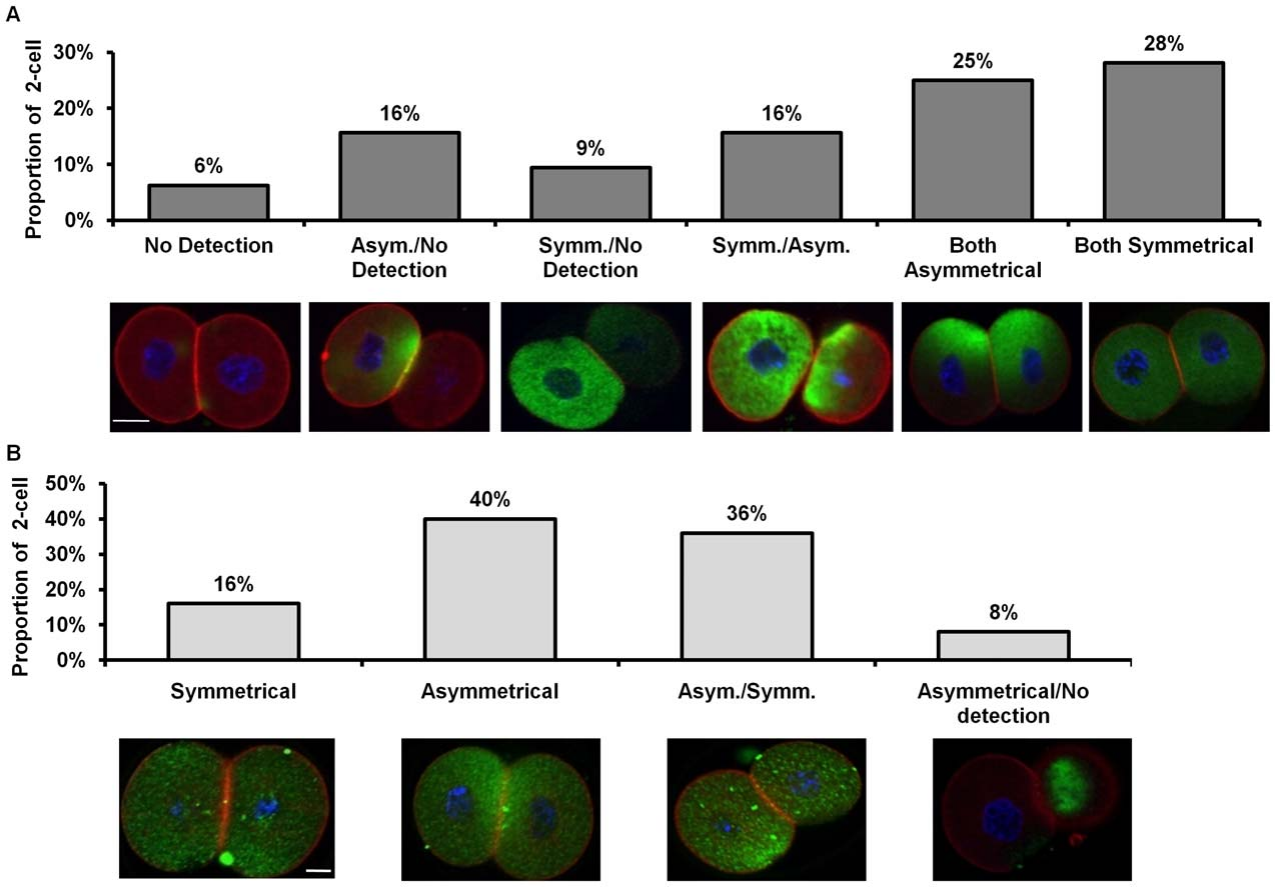
Representative images of SNAI1 and SNAI2 localization at the 2-cell stage. (A) Embryos were categorized based on SNAI1 localization; n = 32, three replicates. (B) Embryos were categorized based on SNAI2 localization; n = 25, three replicates. Red =  Filamentous Actin; Blue = Nuclei; Green  =  SNAI1 (A) or SNAI2 (B). Scale bars  = 10 µM.

As revealed by *Snai1* knock-out studies, SNAI1 plays a role in neural crest differentiation during mouse development [10]. *Snai2* knock-out mice, alternatively, are able to survive to birth [15]. Although there is no preimplantation lethality in the *Snai1* or *Snai2* knock-out, the potential does exist that oogenetic proteins are sufficient to maintain viability past the preimplantation stage in the *Snai1* and *Snai2* nulls. Whole-mount immunofluorescence and confocal microscopy were applied to fresh flushed embryos at each preimplantation developmental stage and revealed a unique distribution pattern. SNAI1 localization appears in discrete fluorescent foci in cortical regions throughout the zygote (Figure 2A). SNAI2 localization is symmetrical however, there are areas with increased intensity throughout the zygote (Figure 2B). SNAI1 and SNAI2 protein immunofluorescence revealed a variety of distribution patterns at the 2-cell stage including an asymmetrical localization within individual blastomeres. In 6% of 2-cell embryos, SNAI1 was not detectable, while in 94% of 2-cell embryos SNAI1 was detectable but displayed variable localization patterns that consisted of either symmetrical or asymmetrical localization patterns within one or both blastomeres of a 2-cell embryo (Figure 3A). 16% of 2-cell embryos displayed a pattern where SNAI1 was asymmetrical in one blastomere and not expressed in the second blastomere, 9% displayed symmetrical distribution in one blastomere and no detection in the second, 16% displayed an asymmetrical and symmetrical pattern, 25% both asymmetrical and 28% where both blastomeres displayed symmetrical distribution (Figure 3A). Furthermore, SNAI1 was consistently detectible in the cytoplasm, however although SNAI1 is a transcription factor, nuclear localization was not commonly observed.

In contrast, SNAI2 was localized to both the cytoplasm and nucleus in positive blastomeres throughout preimplantation development. SNAI2 also displayed both asymmetrical and symmetrical distribution patterns at the 2-cell stage (Figure 3B). 16% of 2-cell embryos displayed symmetrical cytoplasmic localization in both blastomeres, in 40% both blastomeres were asymmetrical and 36% where both blastomeres displayed symmetrical distribution and weak localization to the nuclei. 8% of 2-cell embryos displayed a pattern where in one blastomere SNAI2 was asymmetrical, localized to one pole of the blastomere but not expressed in the nucleus or in the second blastomere (Figure 3B)

**Figure 4:**
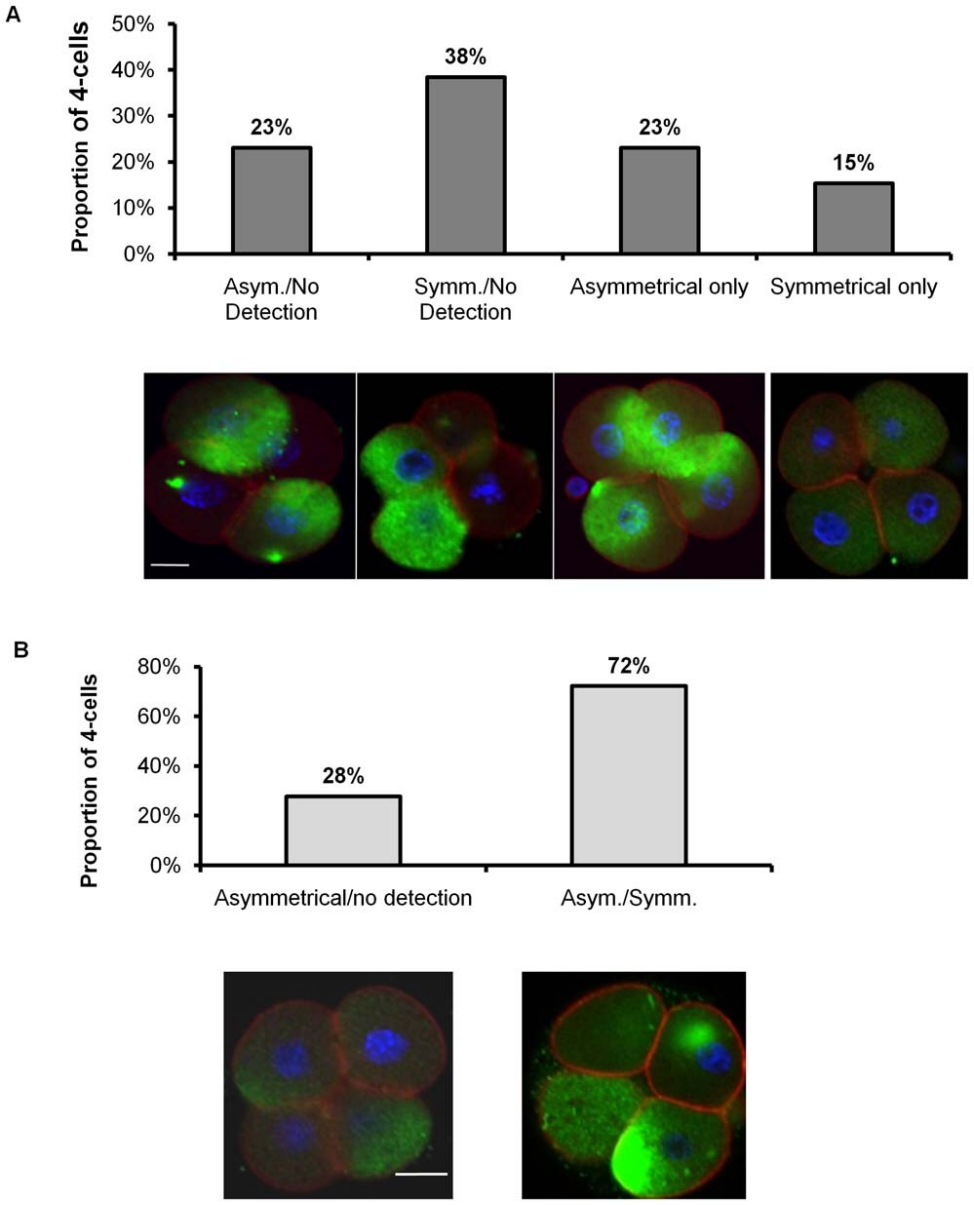
Representative images of SNAI1 and SNAI2 localization at the 4-cell stage. (A) Embryos were categorized based on SNAI1 localization; n = 15, three replicates. (B) Embryos were categorized based on SNAI2 localization; n = 18, three replicates. Red =  Filamentous Actin; Blue = Nuclei; Green  =  SNAI1 (A) or SNAI2(B). Scale bars  = 10 µM.

Detection of SNAI1 and SNAI2 at the 4-cell stage also revealed a variety of distribution patterns, including asymmetrical localization within individual blastomeres (Figures 4A and 4B). Localization patterns included 4-cell embryos expressing SNAI1 in all blastomeres, 3 of the 4 blastomeres, or only 2 blastomeres (Figure 4A).

**Figure 5:**
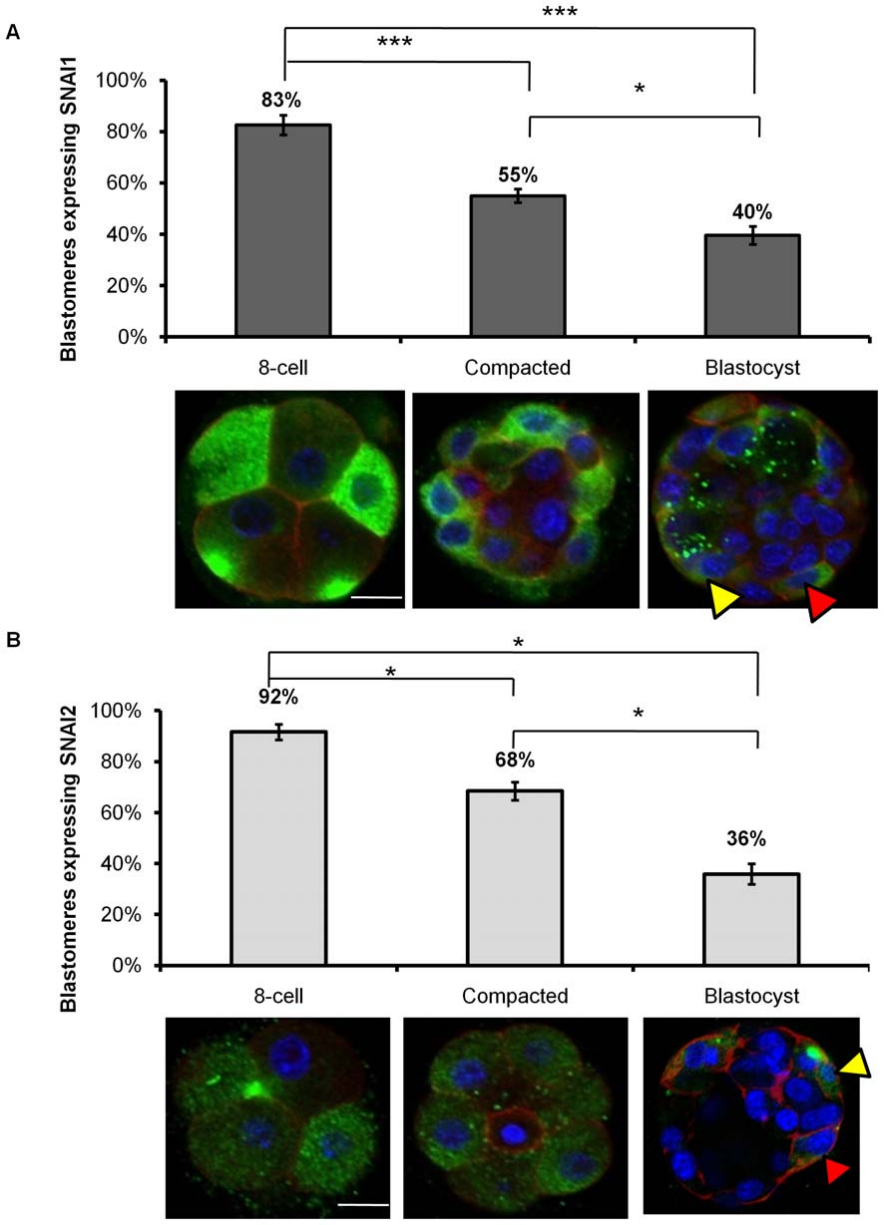
Representative images of SNAI1 and SNAI2 localization at the 8-cell, compacted embryo and blastocyst stage. (A) SNAI1 protein expression pattern at the 8-cell, compacted embryo, and blastocyst stages. The number of blastomeres expressing SNAI1 versus the total number of blastomeres at each stage was counted. 8-cell (n = 16); Compacted embryo (n = 10); Blastocyst (n = 13); *  =  p<0.05; *** =  p<0.001, ±S.E.M. Red =  Filamentous Actin; Blue = Nuclei; Green  =  SNAI1. Scale bars  = 10 µM. (B) SNAI2 protein expression pattern at the 8-cell, compacted embryo, and blastocyst stages. The number of blastomeres expressing SNAI2 versus the total number of blastomeres at each stage was counted. 8-cell (n = 20); Compacted embryo (n = 12); Blastocyst (n = 10); * =  p<0.05, ±S.E.M. Red =  Filamentous Actin; Blue = Nuclei; Green  =  SNAI2. Scale bars  = 10 µM.

SNAI1 and SNAI2 were detected in the majority of blastomeres at the 8-cell stage (Figures 5A and 5B respectively). SNAI1 was either asymmetrically expressed or symmetrically expressed within an individual 8-cell blastomere, however this pattern was consistent across all 8-cell embryos. Asymmetrical expression of SNAI2 was also observed within an individual 8-cell stage blastomere, however symmetrically distributed SNAI2 was the most common pattern observed (Figure 5B). In the compacted embryo, SNAI1 and SNAI2 proteins become confined to the outer cells only and the inner cells no longer displayed SNAI1 or SNAI2 immunofluorescence (Figures 5A and 5B). This localization pattern of SNAI1 and SNAI2 persists throughout development resulting in the detection of SNAI1 and SNAI2 only in the TE of the blastocyst (Figures 5A and 5B). Cell counts performed at the 8-cell, compacted embryo and blastocyst stage revealed the percentage of SNAI1 and SNAI2 positive cells significantly decreased as embryos progressed to the blastocyst stage. As the blastocyst matures and becomes fully expanded, SNAI1 and SNAI2 were decreasingly detected in all TE cells (filled yellow arrow indicates positive staining for SNAI1 (Figure 5A) and SNAI2 (Figure 5B); filled red arrow indicates negative SNAI1 TE cell (Figure 5A) and negative SNAI2 TE cell (Figure 5B).

### Validation of SNAI1 and SNAI2 Antisera

Due to the unique nature of the protein localization patterns we report in our study, we were especially vigilant regarding the specificity of the antisera employed in this study. Antibody peptide pre-absorption controls were conducted using preimplantation embryos. Detection of SNAI1 and SNAI2 was lost in embryos incubated with peptide pre-absorbed antisera compared to embryos incubated in non-pre-absorbed antisera alone (Figures S1A and S1B, respectively). In addition, we knocked down SNAI1 and SNAI2 by microinjecting siRNA into 1-cell embryos targeting either *Snai1* or *Snai2*. SNAI1 and SNAI2 were distinctly knocked down but were still weakly detectable in the siRNA injected embryos. This reduction of SNAl1 and SNAI2 fluorescence further verifies the specificity of the antisera employed in this study (Figures S1C and S1D). We next confirmed the localization patterns of these antisera in published cell models using the same antisera. SNAI1 and SNAI2 immunofluorescence applied to NIH 3T3 cells demonstrated a normal (ie nuclear and cytoplasmic distribution) SNAI1 and SNAI2 localization compared to previously published data (Figures S2A and S2B respectively; (S1C is the negative control)) [17]. We also applied Western Blot analysis, which revealed single and appropriate sized bands for SNAI1 and SNAI2 (Figure S2D). Furthermore, a peptide pre-absorption assay was also performed for the Western blot analysis revealing that the SNAI1 and SNAI2 bands were no longer detectible using pre-absorbed antisera (Figure S2D). These experiments conclusively show that the SNAI1 and SNAI2 antisera employed in this study are specific for SNAI1 and SNAI2, respectively.

## Discussion

This study sought to characterize the expression of the *Snai1* and *Snai2* throughout preimplantation development. Our results have revealed a novel, asymmetrical protein distribution of SNAI1 and SNAI2 within the early preimplantation embryo. *Snai1* and *Snai2* transcripts are detectible throughout preimplantation development. SNAI1 and SNAI2 have variable expression at the 2-cell and 4-cell stage including asymmetrical and symmetrical localization within individual blastomeres. SNAI1 and SNAI2 are detected in the majority of blastomeres at the 8-cell stage, however, protein localization varies between blastomeres within a single embryo. SNAI1 and SNAI2 are then localized to the outer cells in the compacted embryo and are localized only in TE cells of the blastocyst.

*Snai1* pattern of mRNA accumulation does not follow the general pattern of mRNA transcript expression throughout mouse preimplantation development where oogenetic transcripts are largely degraded by the 2-cell stage and then replaced by embryonic expression that drives transcript accumulation after the maternal-to-zygotic transition occurs [18]. This expression pattern supports a novel role for SNAI1 during the maternal-to-zygotic transition (MZT), as its expression is linked closely with the onset of the MZT. This is further supported by studies conducted in zebrafish that demonstrate an increase in *Snai1* transcript during the onset of MZT [19]. *Snai2* transcripts do decrease at the 2-cell stage and therefore more closely follow the typical MZT pattern of mRNA transcript abundance during mouse preimplantation development.

Asymmetrical protein localization in early embryonic blastomeres has been reported by Antczak and Blerkhome, 1997. They reported that Leptin and STAT3 were both asymmetrically expressed in the oocyte and after fertilization, were subsequently distributed unevenly to daughter blastomeres at the 4-cell and 8-cell stage. Consequently, Leptin and STAT3 became distributed in outer cells of the compacted embryo and then in select cells of the TE, leading to the hypothesis that Leptin and STAT3 distribution may contribute to lineage segregation. SNAI1 and SNAI2 are also asymmetrically localized, however, unlike Leptin and STAT3, are expressed in all cells of the 8-cell stage and are not asymmetrically expressed in the outer cells of the compacted embryo or TE of the blastocyst. One similarity is that SNAI1 and SNAI2, like STAT3 and Leptin are expressed in certain cells of the TE in the expanded blastocyst, but not all. It would be interesting to determine if STAT3 and Leptin expression coincided with SNAI1 and SNAI2 expression in the blastocyst [20]. In other vertebrates and invertebrates asymmetrical protein expression is more common (ex. Xenopus) [21]. Asymmetrical expression of proteins in other species plays a role in differentiation by the allocation of that protein to distinct cell lineages through subsequent cellular divisions [21]. Lineage tracing studies would have to be performed to determine segregation of SNAI1 and SNAI2 between the 2-cell to 4-cell division in the mouse embryo. Furthermore, it has been demonstrated that each blastomere at the 8-cell stage retains the ability to contribute to all cell lineages of the early embryo and thus is referred to as being “totipotent” [1]. Our results indicate that blastomeres at the 2-cell, 4-cell and 8-cell stages do not all localize SNAI1 or SNAI2 equally and thus while all early blastomeres may display totipotence, they are not all equivalent. This variable pattern of SNAI1 and SNAI2 localization may be regulated by the cell cycle as early blastomeres undergo asynchronous cell divisions. However, the asymmetrical SNAI1 and SNAI2 localization patterns within individual blastomeres cannot result from cell cycle variation between blastomeres and likely results from cytoskeletal based mechanisms within the cell.

Our data do not allow us to conclude that SNAI1 or SNAI2 are active contributors to cell fate decisions during preimplantation development, but certainly SNAI1 and SNAI2 are markers of cell fate decisions as their distribution patterns reflect the cell lineage decisions that occur during the first week of development. It is intriguing to propose that blastomeres displaying variable SNAI1 and SNAI2 localization patterns may divide in varying planes, which would produce an uneven inheritance of SNAI1 or SNAI2 to the daughter blastomeres, and thus contribute to cell fate decisions in the early embryo. Alternatively, blastomeres that have symmetrical distribution of SNAI1 or SNAI2 may divide radially, where both daughter blastomeres would inherit SNAI1 or SNAI2. Our studies will pursue the determination of the role(s) that SNAI1 and SNAI2 play in preimplantation development and possible cell fate decisions.

It has been proposed that blastomeres at the 2-cell stage are fated to contribute to the embryonic or abembryonic portion of the embryo [22]. Furthermore, studies have suggested that 4-cell blastomeres are not equally pluripotent due to the manner in which 2-cell blastomeres cleave resulting in an uneven distribution of products across the blastomeres [23]. Our results demonstrate that SNAI1 and SNAI2 are unevenly distributed at the 2-cell stage and the 4-cell stage, however, careful analysis has demonstrated that SNAI1 and SNAI2 can be found in all blastomeres at the 4-cell stage regardless of blastomere position. Furthermore, SNAI1 and SNAI2 are expressed in all blastomeres at the uncompacted 8-cell stage and are expressed in all outer cells of the compacted embryo and in both the abembryonic and embryonic portions of the TE in the expanded blastocyst.

This study reveals asymmetrical distribution of SNAI1 and SNAI2 as early as the 2-cell and 4-cell stage of preimplantation development, far earlier than previously thought. Not only are SNAI1 and SNAI2 asymmetrically distributed within early cleavage stage blastomeres but their expression is lost in the ICM coincident with cell lineage specification and the formation of outer and inner cell lineages in the early embryo. This may indicate that SNAI1 and SNAI2 may contribute to cell polarization and epithelial cell differentiation. Collectively, this study provides novel insight into the potential role of SNAI1 and SNAI2 in development and cancer metastasis.

## Materials and Methods

### Collection of Preimplantation Embryos

Mouse embryos were obtained from random-bred MF1 females superovulated with pregnant mare's serum gonadotropin (PMSG) and human chorionic gonadotropin (hCG), and mated with CD1 males. Successful mating was determined the next morning (day 1) by the presence of a vaginal plug. Embryos were collected at specified times following hCG injection, which correspond to appropriate cleavage stages: 1-cell inseminated zygotes, 18-hr post-hCG; 2-cell, 48 hr; 4-cell, 60 hr; 8-cell, 65–68 hr; compacted embryo, 80–85 hr and blastocysts, 90 hr. Embryos were then washed in 50 µl M2 media and frozen in groups of 20 for quantitative RT-PCR analysis or fixed for whole mount immunofluorescence detection of SNAI1 and SNAI2 proteins.

### Mouse Fibroblast NIH 3T3 Cell Culture

NIH 3T3 cells were plated in 6-well culture dishes at a density of 5×10^3^ cells/cm^2^. Cells were cultured in complete medium supplemented with 10% fetal bovine serum (Sigma, St Louis, MO), 1% Antibiotics (Sigma) in DMEM. Cells were trypsinized with 1X trypsin for 1 min at 37°C, harvested in 3–5 ml complete medium and centrifuged for 5 min at 4000×g. Cells were re-suspended in 10ml complete medium and plated on chamber slides for immunofluorescence analysis or on 15 cm plates for protein analysis. Cells were cultured for 2 days until they reached confluence and fixed for immunofluorescence or lysed for whole cell lysate collection.

### Quantitative RT-PCR

Real-time RT-PCR was conducted to determine the mRNA profile of *Snai1* and *Snai2* at the 1-cell, 2-cell, 4-cell, 8-cell, compacted embryo and blastocyst stages. Prior to RNA extractions, Luciferase mRNA, an exogenous control, was added to the samples. Total RNA was extracted from pools of 20 embryos using the PicoPure kit (Arcturus, Molecular Device, Sunnyvale, CA). Reverse Transcription reaction was carried out using Sensiscript reverse transcriptase (Qiagen, Mississauga, ON). The samples were incubated in 10x buffer, RNAse inhibitor, dNTPs and random primers at 37°C for 1 hour. Quantitative PCR reactions were performed using the BioRad Chromo4 detection system (BioRad, Mississauga, ON). PCR was carried out in 25 µl reactions containing 12.5 µl Multiplex Universal PCR Master Mix (2X concentration solution optimized for multiplex reactions, BioRad), 1.25 ul of SNAI1 and SNAI2 primer/probe sets provided by Biosearch Technologies (Novado, CA), or SYBR Green (Invitrogen, Burlington, ON) and 5 µl of appropriate dilution of cDNA (0.1 embryo/µl), and 6.25 µl of water. *Snai1* primer sequence 5′-CATCCTCGCTGGCATCTTCC-3′; 3′-GAGAGCCAAGCAGGAACCAG-5′; *Snai2* primer sequence 5′-CTTTACCCAGTGGCCTTTCTC-3′; 3′-CCACAGATCTTGCAGACACAA-5′.

### Whole-Mount Immunofluorescence

Protein localization was investigated using whole-mount immunofluorescence methods for both SNAI1 and SNAI2 throughout preimplantation development. Embryos were fixed in 2% paraformaldehyde in PBS and then incubated in primary rabbit polyclonal anti-SNAI1 (ab 17732, Abcam, Cambridge, MA and gift from Dr. Paul Wade and Dr. Archana Dhasarathy) or rabbit polyclonal anti-SNAI2 antibodies (ab27568, Abcam). Negative controls were incubated in antibody dilution buffer alone. NIH 3T3 cells were fixed in 4% paraformaldehyde in PBS for 20 min. Cells were then washed and incubated with primary SNAI1 or SNAI2 antibody. Negative control cells were incubated in antibody dilution buffer alone. Antibody specificity was determined by conducting immuno-pre-absorption assays with either a SNAI1 peptide (Abcam) for the SNAI1 antiserum or a SNAI2 peptide (Abcam) for the SNAI2 antiserum. Fixed embryos were then incubated in either SNAI1 or SNAI2 antisera alone, or in the pre-absorption antibody solution. Embryos and cells were examined by confocal microscopy using an Olympus Fluoview 1000 laser scanning Confocal Microscope (Olympus, Canada). Z-stack images were taken of the embryos and used to count total cell number and SNAI1 or SNAI2 positive cells.

### Western Blot

NIH 3T3 cells were lysed, on ice, using RIPA buffer with Protease inhibitors. Cells were then scraped into the RIPA buffer and homogenized by repeated pipetting. Cell lysate was removed and placed into a 1.5 ml tube and centrifuged at 4000×g for 5 min. The supernatant was removed and stored at −80°C. Protein Quantification was performed using D_C_ Protein Assay kid (BioRad) and a spectrophotometer. A 12% acrylamide stacking gel was used and 40 mg of protein was loaded into each lane. Antiserum dilutions employed included SNAI1 anti-serum (1∶1000); SNAI2 antiserum (1∶2000); and secondary antibody (Anti-Rabbit IgG HRP-linked (Cell Signalling, Danvers, MA) at 1∶10000). Antiserum pre-absorption assay was performed prior to membrane incubation.

### Microinjection of siRNAs

Microinjection was performed under an inverted microscope using a mechanical micromanipulator (Leica, Richmond Hill, ON, CA) attached to Picoinjector PLI-100 (Harvard Apparatus, Saint-Laurent, PQ, CA). 1-cell embryos were injected with either negative control (Invitrogen) or siRNA duplexes targeting *Snai1* or *Snai2.* Microinjection of embryos was performed according to a standard procedure. One-cell embryos were placed on a concave watch glass and into KSOMaa medium under light mineral oil. A holding pipette (Conception Technologies, San Diego, CA) was used to keep the one-cell embryos stationary during manipulation. An injection pipette loaded with siRNA solution was inserted into the cytoplasm of each zygote followed by the microinjection of approximately 20 pl of 20 µM of dsRNA. After microinjection, embryos were cultured in KSOMaa medium as described above for 48 hours and fixed at the 4–8 cell stage and used for whole-mount immunofluorescence as described above.

### Statistics

Quantitative RT-PCR and whole-mount immunofluorescence were performed in triplicate using pools of embryos at each stage collected from 3 groups of mice. Data was analyzed using the SigmaStat® 3.5 (Jandel Scientific Software, San Rafael, CA, USA). Upon acceptance of normality tests, the data was analyzed using a One-way ANOVA followed by a Bonferroni multiple comparison test. If the data failed the normality test, it was analyzed using an ANOVA on ranks, followed by a Dunn's multiple comparison test.

## Supporting Information

Figure S1 Antisera validation in preimplantation embryo. (A and B) Antibody pre-absorption assays revealed the specificity of the SNAI1 (A) and SNAI2 (B) antisera. Embryos incubated in antisera pre-absorbed with SNAI1 or SNAI2 peptide displayed comparable levels of SNAI1 or SNAI2 expression with negative controls. (C and D) Negative siRNA or siRNA targeting Snai1 or Snai2 was microinjected into 1-cell embryos. Whole-mount immunofluorescence was applied detecting SNAI1 (F) and SNAI2 (G) in control and knock-down embryos. SNAI1 (F) and SNAI2 (G) expression was significantly down regulated in the embryos that were microinjected with siRNA targeted to Snai1 or Snai2.
(5.47 MB TIF)

Figure S2 Antisera validation in NIH3T3 cells. (A and B) SNAI1 and SNAI2 antibodies were used to detect SNAI1 (A) and SNAI2 (B) in NIH3T3 cells (C- no primary control). This experiment revealed that these antisera replicated the published localization pattern of SNAI1 and SNAI2 in NIH3T3 cells. (D) Western Blot analysis was performed to determine the specificity of the antisera. Single protein bands for both SNAI1 and SNAI2 were detected at the expected molecular weight for each protein. Protein bands were no longer detected when membranes were incubated in pre-absorbed SNAI1 and SNAI2 antisera. Red = Filamentous Actin; Blue = Nuclei; Green  =  SNAI1 or SNAI2. Scale bars  =  10 µM.
(3.03 MB TIF)
