# Supplementary figures and images for: SNAI1 and SNAI2 Are Asymmetrically Expressed at the 2-Cell Stage and Become Segregated to the TE in the Mouse Blastocyst
Source: PLoS One. 2009 Dec 31;4(12):e8530. doi: 10.1371/journal.pone.0008530 (PMC2796167; doi:10.1371/journal.pone.0008530)

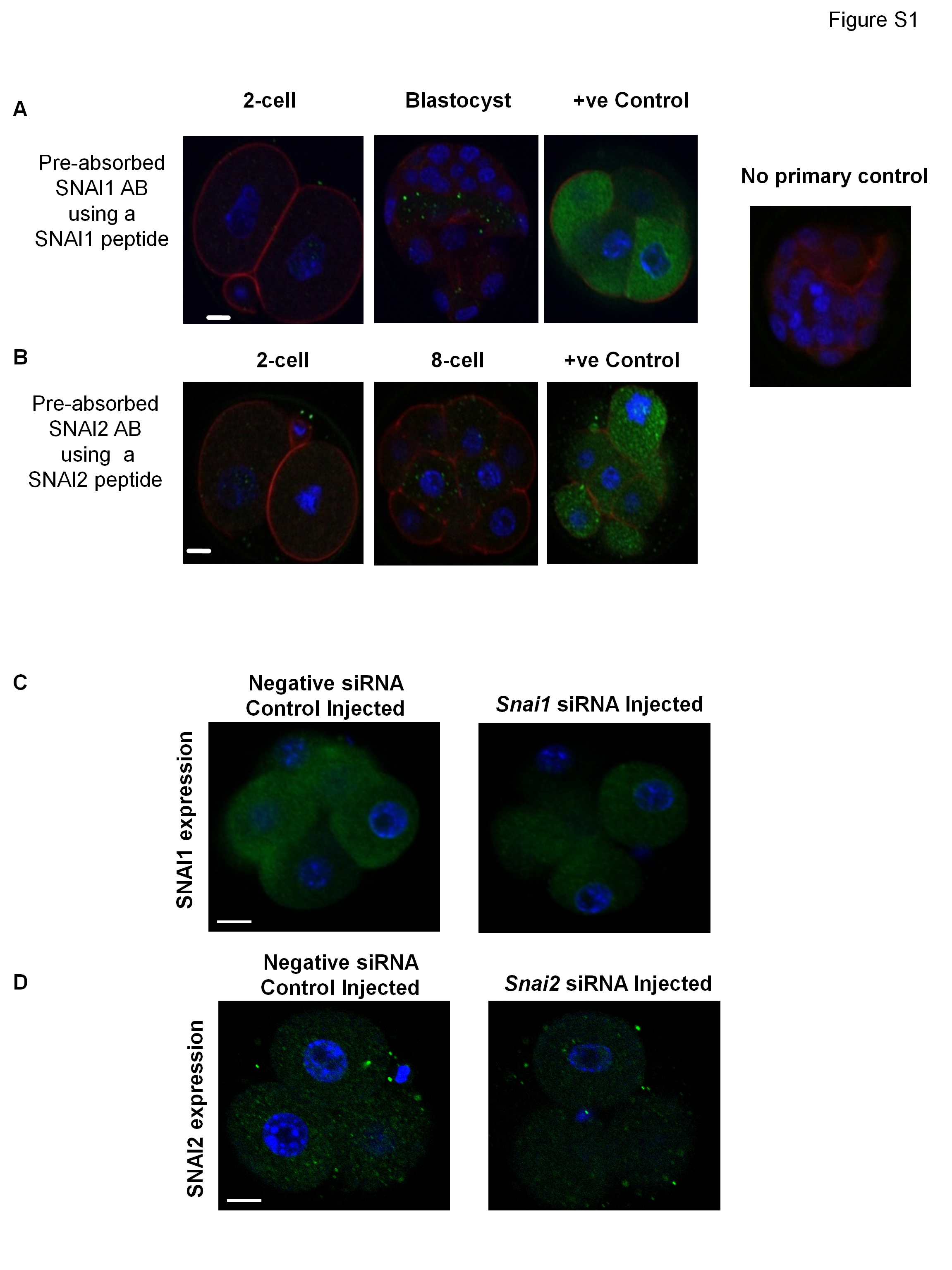

Supplement: Figure S1 — Antisera validation in preimplantation embryo. (A and B) Antibody pre-absorption assays revealed the specificity of the SNAI1 (A) and SNAI2 (B) antisera. Embryos incubated in antisera pre-absorbed with SNAI1 or SNAI2 peptide displayed comparable levels of SNAI1 or SNAI2 expression with negative controls. (C and D) Negative siRNA or siRNA targeting Snai1 or Snai2 was microinjected into 1-cell embryos. Whole-mount immunofluorescence was applied detecting SNAI1 (F) and SNAI2 (G) in control and knock-down embryos. SNAI1 (F) and SNAI2 (G) expression was significantly down regulated in the embryos that were microinjected with siRNA targeted to Snai1 or Snai2. (5.47 MB TIF) [file pone.0008530.s001.tif]

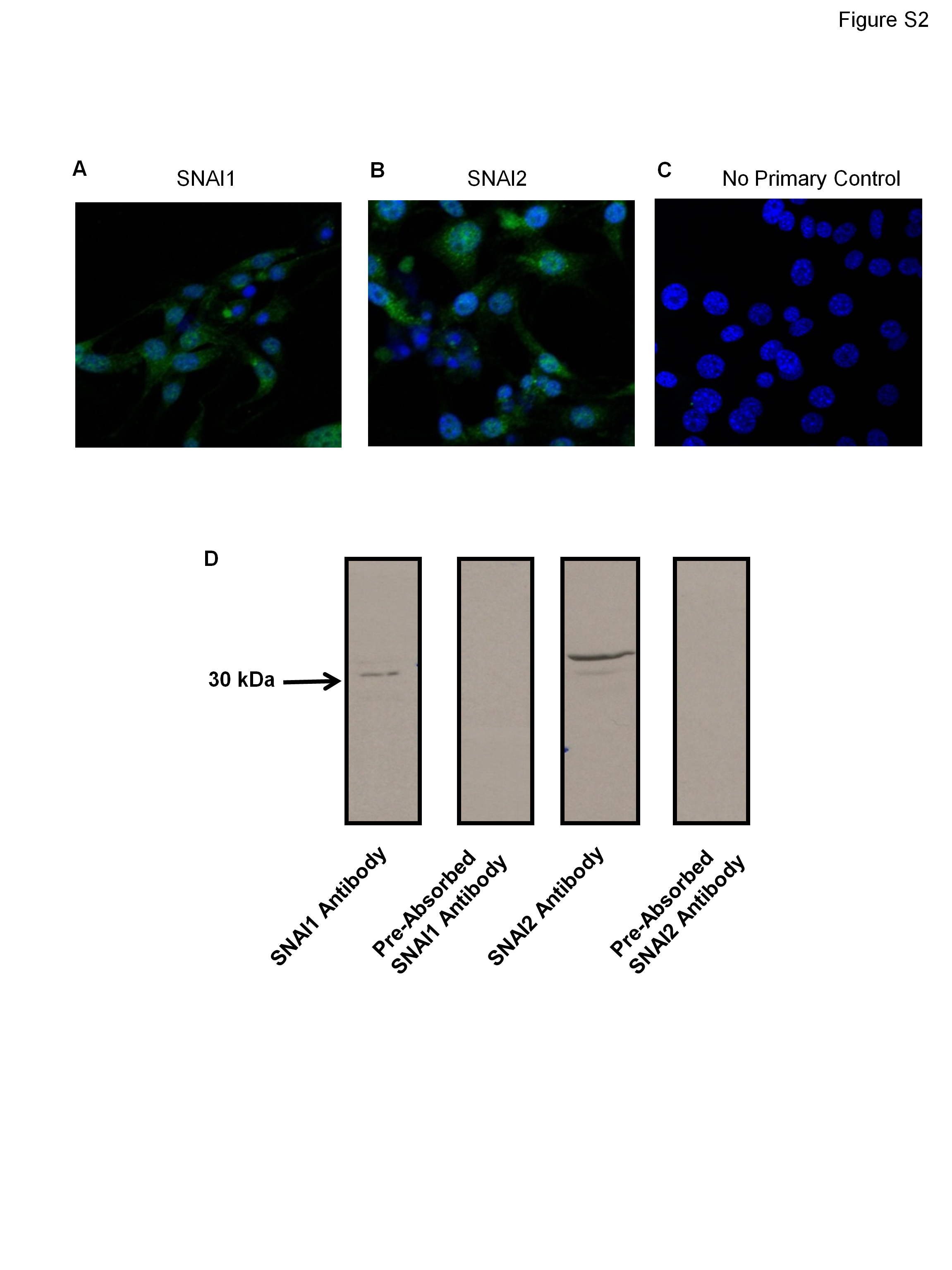

Supplement: Figure S2 — Antisera validation in NIH3T3 cells. (A and B) SNAI1 and SNAI2 antibodies were used to detect SNAI1 (A) and SNAI2 (B) in NIH3T3 cells (C- no primary control). This experiment revealed that these antisera replicated the published localization pattern of SNAI1 and SNAI2 in NIH3T3 cells. (D) Western Blot analysis was performed to determine the specificity of the antisera. Single protein bands for both SNAI1 and SNAI2 were detected at the expected molecular weight for each protein. Protein bands were no longer detected when membranes were incubated in pre-absorbed SNAI1 and SNAI2 antisera. Red = Filamentous Actin; Blue = Nuclei; Green = SNAI1 or SNAI2. Scale bars = 10 µM. (3.03 MB TIF) [file pone.0008530.s002.tif]
